# Lipedema: exploring pathophysiology and treatment strategies – state of the art

**DOI:** 10.1590/1677-5449.202400252

**Published:** 2025-01-20

**Authors:** Fabio Kamamoto, Jaqueline Munaretto Timm Baiocchi, Bernardo Nogueira Batista, Renan Diego Américo Ribeiro, Débora Aparecida Oliveira Modena, Vitor Cervantes Gornati

**Affiliations:** 1 Instituto Lipedema Brasil, São Paulo, SP, Brasil.; 2 A.C. Camargo Cancer Center, São Paulo, SP, Brasil.; 3 Hospital Sírio Libanês, Unidade de Mama, São Paulo, SP, Brasil.; 4 Universidade de São Paulo – USP, Faculdade de Medicina de Ribeirão Preto – FMRP, São Paulo, SP, Brasil.; 5 Prime Care Medical Complex, São Paulo, SP, Brasil.

**Keywords:** lipedema, lipolymphedema, lipodystrophy, adipose tissue dysfunction, abnormal fat deposits, lipedema, lipolinfedema, lipodistrofia, disfunção do tecido adiposo, depósitos anormais de gordura

## Abstract

Lipedema is characterized by abnormal fat deposition in areas such as the arms, hips, buttocks, and thighs, sparing the hands and feet. Symptoms include pain, bruising, edema, and subcutaneous nodules, which resist traditional interventions such as diet and exercise. Despite increasing recognition, comprehensive understanding, including pathophysiological, clinical, and therapeutic aspects, has not been fully achieved. This review aims to fill gaps in knowledge of this field, to support more informed management of lipedema. This narrative review provides a deeper understanding of lipedema treatment, addressing pathophysiology and therapeutic options. The data reveal advances in knowledge, especially regarding conservative and surgical treatments, focusing on improving quality of life. However, scientific evidence supporting the safety and efficacy of various treatments is lacking. Additional research is needed to ensure safety and to enhance efficacy of management of this complex condition.

## Resumo

O lipedema é caracterizado pela deposição anormal de gordura em áreas como braços, quadris, nádegas e coxas, poupando as mãos e os pés. Os sintomas incluem dor, hematomas, edema e nódulos subcutâneos, que resistem a intervenções tradicionais, como dieta e exercício. Apesar do crescente reconhecimento, a compreensão abrangente, incluindo aspectos fisiopatológicos, clínicos e terapêuticos, ainda não está totalmente consolidada. Esta revisão tem como objetivo preencher lacunas no conhecimento da área, visando um manejo mais informado do lipedema. Foi possível proporcionar uma compreensão mais aprofundada do tratamento do lipedema, abordando a fisiopatologia e as opções terapêuticas. Os dados revelaram avanços no conhecimento, principalmente no que diz respeito aos tratamentos conservadores e cirúrgicos, com foco na melhoria da qualidade de vida. No entanto, continuam faltando evidências científicas que apoiem a segurança e a eficácia de vários tratamentos. A necessidade de pesquisas adicionais é enfatizada para garantir a segurança e aumentar a eficácia do manejo dessa condição complexa.

## INTRODUCTION

### History

The earliest reports of dysfunction associated with abnormal fat deposits date back to 1910, when Irving Phillips Lyon analyzed patients with excess fat, naming the condition "adipose" and identifying "lipomatosis" as abnormal adipose tissue growth.^1-3^

His description was a milestone for differentiating types of subcutaneous adipose tissue dysfunctions, enabling investigations and the first detailed description of lipedema in the United States, published in 1940 by cardiovascular physicians Edgar Allen and Edgar Alphonso Hines Jr.^2-4^

Since then, lipedema has been recognized as abnormal fat deposition in the arms, hips, buttocks, and thighs, notably sparing the hands and feet. Symptoms include pain, a propensity for bruising, edema, firm subcutaneous nodules of adipose tissue, and resistance of the fat to traditional diets and exercise interventions.^2-4^

Its clinical complexity poses challenges to understanding, making differential diagnosis from lymphedema and/or obesity difficult. According to Fife et al.,^5^ 10% to 20% of patients referred to clinics for lymphedema treatment ultimately receive a lipedema diagnosis. Furthermore, up to 50% of patients with lipedema are overweight or obese, but have a low prevalence of metabolic diseases such as diabetes, dyslipidemia, and hypertension.^6^

Despite having been described for more than 80 years, lipedema was only included in 2019 in the 11th International Classification of Diseases (ICD-11) as a separate clinical entity in the category "Certain noninflammatory disorders of subcutaneous fat", with the following description: "Lipedema is characterized by diffuse 'fatty' swelling with no depressions, usually confined to the legs, thighs, hips, and arms. It may be mistaken for lymphedema."^7^

### Epidemiology

Prevalence in the general population varies from 0.06% to 10–11%, reaching higher rates in some countries, such as Germany, where it affects up to 39% of women. In Brazil, the prevalence of lipedema is estimated to be 12.3% among Brazilian women. With approximately 100.5 million women in Brazil in 2021, it can be predicted that 8.8 million of them may have symptoms suggestive of lipedema.^5-8^

In 2021, Mattis Bertlich and colleagues presented a case report of lipedema in a male individual. During the literature review, they highlighted the remarkable scarcity of documented records of lipedema in men, and when mentioned, they were often associated with specific medical conditions; only two cases were diagnosed as idiopathic lipedema. In summary, fewer than 10 cases of lipedema in men have been documented in the literature to date.^9-11^

In recent years, lipedema has gained recognition among both healthcare professionals and the general population. This increased visibility is attributable to online dissemination of information and growing medical attention devoted to this condition. However, comprehensive knowledge of lipedema, including its pathophysiology, clinical aspects, and conservative and surgical treatment modalities, is not yet fully consolidated in the healthcare field.

This article presents a narrative review to deepen understanding of lipedema, addressing its pathophysiology, clinical aspects, and therapeutic options. The main objectives are to fill gaps in existing knowledge, consolidate information, and promote awareness of this condition.

## METHODOLOGY

### Protocol and eligibility criteria for study identification

This comprehensive narrative review of lipedema included clinical trials with no restrictions on date, sample size, or language. Relevant studies were identified from databases such as NCBI/PubMed, Latin American and Caribbean Health Sciences Literature, Medline, Embase, CINAHL, and the Cochrane Library. The search strategy employed Medical Subject Headings (MeSH) and was adapted to suit the requirements of each database.

### Study selection

The search strategy for the scientific databases was implemented by two researchers (DAOM and JMTB), with a third senior researcher (BNB) resolving disagreements. Duplicates were eliminated using Mendeley Reference Manager®. Studies were initially screened by title and then by abstract and those relevant to the theme were accessed in full for evaluation and inclusion.

## LITERATURE REVIEW

### Physiopathological aspects of lipedema

The cause of lipedema remains unclear, and various hypotheses have been suggested. These include genetic predispositions and hormonal changes, particularly the influence of estrogen, leading to abnormalities in adipocyte growth and differentiation and microvascular dysfunction in lymphatic and blood vessels.^8-10^

### Genetic predisposition

Lipedema is recognized as a condition with a genetic predisposition, with a considerable likelihood of autosomal dominant inheritance.^9-12^

According to Paolacci et al.,^12^ a positive self-reported family history is notable in 64% of women affected by lipedema, corroborated by research that identified affected family members. Lipedema can be associated with other medical conditions, resulting in development of syndromic lipedema. Syndromes identified include Sotos syndrome, which is linked to NSD1 gene mutations and presents with features such as normal intelligence, insulin-dependent diabetes, asthma, and lipedema. Despite progress, a specific gene directly linked to lipedema has not been identified.^9,12-14^

### Hormonal factors

Lipedema predominantly affects female individuals, with onset coinciding with hormonal fluctuations, notably in estrogen. Studies indicate that changes in the distribution pattern of alpha and beta estrogen receptors disrupt their signaling pathways, resulting in increased estrogen release.^15-17^ This hormonal surge initiates a cascade of metabolic changes, including activation of PPARγ receptors, increased uptake of glucose and free fatty acids, and increased angiogenesis. Simultaneously, there are reductions in lipolysis and formation of new mitochondria. These metabolic shifts directly influence fat storage and adipose tissue growth. When these processes become dysregulated, adipocytes accumulate excess fat, contributing to the characteristic fat deposition observed in lipedema.^15-17^

### Dysfunction in adipocyte hyperplasia and hypertrophy

Adipocyte hypertrophy is the primary manifestation of lipedema, although hyperplasia may also occur under certain conditions. This hypertrophy results from a complex interaction of genetic factors, hormonal dysregulation (notably influenced by estrogen), and inflammatory processes, leading to adipogenesis dysfunction.^18-22^

Studies by Felmerer et al.,^18^ Kruppa et al.,^20^ and Al-Ghadban et al.^23^ revealed significant adipocyte hypertrophy, interstitial fibrosis, and increased macrophage presence in lipedema.

Vasella et al.^22^ found that lipedema patients had increased expression of the inflammatory protein MIF-1 but not MIF-2, indicating low-grade adipocyte inflammation distinct from obesity.

This difference in inflammation profiles helps explain the unique characteristics of lipedema compared with other adipose tissue-related conditions, such as obesity.

### Primary microvascular dysfunction in the lymphatic and blood capillaries

Al-Ghadban et al.^23^ investigated vascular and lymphatic alterations in lipedema by comparing adipose tissue from nonobese and obese women with lipedema. They observed increased angiogenesis in the dermis and hypodermis, particularly in obese participants, indicating an increase in blood vessel numbers. Lymphatic changes were more subtle but included an increase in vessel size and capillary dilation in obese participants with lipedema.

Development of lipedema is linked to primary microvascular dysfunction, beginning with endothelial cell alterations that disrupt blood and lymphatic flow regulation, leading to edema and a cyclic inflammatory response.^17,24^

Unregulated angiogenesis is characterized by an imbalance in proangiogenic and antiangiogenic factors, resulting in microangiopathy with immature, hemorrhage-prone blood vessels. Increased capillary permeability contributes to abnormal fat expansion and tissue hypoxia.^17,24^

Hemodynamic dysfunction in lipedema affects blood circulation and lymphatic capillary functionality, limiting lymphatic drainage. This issue is worsened by increased free fatty acids and other byproducts from adipocyte hyperproliferation in the interstitial environment.^17,23-25^

Duhon et al.^25^ reported high levels of platelet factor 4 (PF4), associated with lymphedema, in lipedema patients, suggesting that lymphatic system dysfunction is related to adipose tissue expansion in lipedema.

Chachaj et al.^24^ reported that lipedema burdens the lymphatic system even in its early stages, leading to oxidative stress and triggering pathological events such as adipocyte necrosis, release of inflammatory cytokines, and fibrosis. Mechanical and gravitational impacts, such as prolonged standing, also play a role by exerting pressure on blood and lymphatic capillaries, hindering circulation and drainage.

Lipedema, lymphedema, and chronic venous insufficiency (CVI) are distinct yet interrelated conditions affecting the circulatory and lymphatic systems, leading to fluid flow alterations. Lipedema involves symmetrical fat accumulation in the limbs, mainly the legs, which can be painful. Lymphedema results from the accumulation of lymphatic fluid due to a damaged or blocked lymphatic system, causing limb swelling. CVI involves inefficient blood return to the heart, leading to varicose veins, edema, and severe skin changes.^11,17,24^

Lipedema and lymphedema can coexist, complicating diagnosis and management, with CVI potentially exacerbating both conditions owing to decreased venous return efficiency, fluid accumulation, and increased pressure in affected tissues. Accurate diagnosis is crucial to differentiate these conditions to enable appropriate treatment, which may include manual lymphatic drainage, compression stockings, exercise, and invasive interventions in selected cases.^11,17,24^

The pathophysiology of lipedema involves a complex interaction of factors, as recent research reveals, but fully understanding its mechanisms remains challenging, underscoring the need to continue research efforts to elucidate its complexity.

### Clinical presentation of lipedema

Individuals with lipedema present fat distribution with a gynoid body type, resulting in notable disharmony between the upper and lower parts of the body^26-28^ (Table 1).

**Table 1:** Clinical Manifestations of Lipedema.

**Edema in the lower limbs worsening in the afternoon or evening.**	**Edema in the lower limbs related to orthostasis, heat, and/or physical activity.**
Increased sensitivity to spontaneous pain or pain induced by trauma or pressure.	Spontaneous or trauma-induced bruises.
Antalgic gait with misalignment of the leg axis.	Hypermobility and joint stress - osteoarthritis.
Abnormalities in the plantar arch with excessive foot pronation.	Dermal lesions, maceration, infections, and varicosities.
Abnormal distribution of persistent fat; overweight or morbid obesity.	Eating disorders and psychological disturbances.
Unilateral or typically bilateral lymphedema or lympholipedema.	Lower prevalence of diabetes and hypertension, possible aortic stiffness.

### Forms of diagnosis

Diagnosing lipedema is challenging because of the absence of specific markers. Evaluation is therefore predominantly based on medical history and clinical examination.^20,29^

Several elements of patient medical history are crucial, such as age, onset pattern, family history, affected areas, influence of diet, presence of pain, bruising, skin characteristics, impact on daily activities, and subjective understanding of the disease.^20,29^

For screening, the Lipedema Symptom Assessment Questionnaire (LSyQu) is a fundamental auxiliary tool for predicting lipedema. The questionnaire addresses nine self-reported criteria, focusing on individuals’ perceptions of their bodies. It explores whether the patient observes irregularities in the legs, disproportions in the lower body compared with the trunk, and difficulties with weight loss and weight gain in specific areas during hormonal fluctuations, such as the thighs, legs, hips, buttocks, or arms.^30^

Inspection and palpation should be performed during the physical examination, with an emphasis on reports of clinical manifestations, considering the stage and location of lipedema tissue, body mass index (BMI), presence of metabolic disease, and edema, which intensify with progression of the condition. Additionally, Kruppa et al.^20^ reported clinical criteria that should be considered important for diagnosis, as shown in Table 2.

**Table 2:** Clinical Criteria for Lipedema Diagnosis.

**Disproportionate hypertrophy in the limbs: bilateral and symmetrical increase in adipose volume.**
Preservation of hands and feet (cuffing phenomenon): maintenance of normal size in distal extremities, contrasting with increased volume in proximal regions.
Approximately 30% involvement of the arms.
Negative Stemmer’s sign: indicating that the skin fold between the second and third toe does not show thickening and can be lifted.
Feeling heaviness and tension: subjective perception of burden and tension in the affected limbs.
Pain upon pressure and touch: painful sensitivity in response to pressure and physical contact.
Marked tendency to bruise: exaggerated propensity for the occurrence of bruising.
Stable limb circumference: stability in the circumference of the limbs, even with weight loss or calorie restriction.
Worsening of symptoms throughout the day.
Visible telangiectasias and varicose veins: presence of small visible blood vessels and varicose veins around areas of fat deposition.
Skin hypothermia: low skin temperature, suggesting altered vascular response.

Adapted from Kruppa et al.^20^.

**Figure 1:**
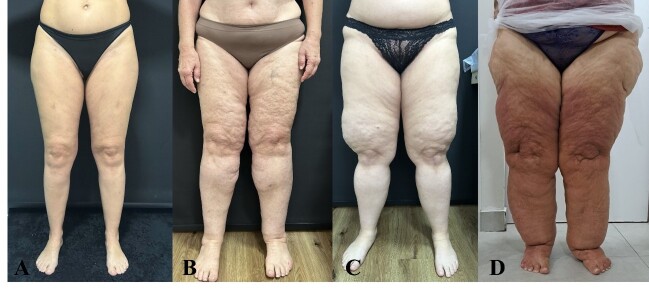
Stages of lipedema according to severity. **(A)** Stage I; **(B)** Stage II; **(C)** Stage III; **(D)** Stage IV. Source: author’s files.

Through meticulous medical history taking and clinical examination, it is possible to categorize lipedema into types, classification of which is intrinsically linked to the distribution of adipose tissue, and into stages, categorization of which is correlated with the severity of the condition^27-29,31^ (Table 3 and Figure 1).

**Table 3:** Types of lipedema according to fat distribution and stages of lipedema according to severity.

**TYPES**	**STAGES**
Type I: accumulation of adipose tissue around the hips and buttocks.	Stage I: smooth skin with small nodules, reversible edema.
Type II: accumulation of adipose tissue spanning from the hips to the knees.	Stage II: irregular or wrinkled skin with walnut-sized nodules, reversible or irreversible edema.
Type III: accumulation of adipose tissue with a hip-to-ankle phenotype.	Stage III: thickened and hardened skin with disfiguring fat deposits, often associated with functional limitations.
Type IV: accumulation of adipose tissue even in the arms.	Stage IV: lipolymphedema, development of lymphedema concomitant with lipedema.
Type V: predominance of fat exclusively in the calf region.	

Adapted from Forner-Cordero et al.^29^.

Diagnosis of lipedema is based on medical history and physical examination, which may include imaging procedures, genetic analyses, and histological analyses, as necessary. Although computed tomography and magnetic resonance imaging have been suggested for distinguishing lymphedema from lipedema, their high costs and limited availability have been limiting factors. As an alternative, recent research has explored the application of high-resolution ultrasound.^29,31^

A pioneering study conducted by Naouri et al.^31^ demonstrated the effectiveness of high-resolution ultrasound for differentiating between lipedema and lymphedema. This study involved examination of the lower limbs at three specific sites: the front of the thigh, the region between the knee and the lateral malleolus, and above the lateral malleolus. Dermal thickness, dermal echogenicity, and the dermo-hypodermal junction were analyzed. The results revealed that dermal thickness, echogenicity, and identification of the dermo-epidermal junction were essentially normal in patients with lipedema, whereas, in patients with lymphedema dermal thickness was increased, there was a marked reduction in dermal echogenicity, making it darker in the image, and the dermo-hypodermal junction was not fully delimited owing to accumulation of edema in the superficial dermis.

Following this study, several others emerged,^32-35^ many of which employ diagnostic criteria published by Amato and colleagues.^36^ This group established cut-off values through a simple and reproducible method to diagnose lipedema in the lower limbs. The criteria established refer to different regions of the legs, with proposed cut-off values for each of them, the pretibial region (anterior leg): 11.7 mm; the frontal thigh region: 17.9 mm; and the lateral leg region: 8.4 mm. These cut-off values were proposed to distinguish between lipedema and individuals in normal conditions, indicating a greater likelihood of lipedema when dermal and subcutaneous tissue thickness measurements exceed these values.^36^

Studies have suggested a possible link between the lipedema phenotype and specific genes, such as the IL-6 (Interleukin-6) gene described by Di Renzo et al.^37^ and the LHFPL6 gene identified by Precone et al.^38^ Other studies, including those of Klimentidis et al.,^39^ have revealed significant associations between the genetic loci VEGFA and GRB14-COBLL1 and lipedema.

Another noninvasive approach for assessing and diagnosing lipedema is dual-energy X-ray absorptiometry (DXA), used to measure body composition and analyze fat mass distribution. Results highlight the utility of this modality, providing objective and measurable criteria that complement subjective clinical criteria for diagnosis and staging of lipedema. By identifying the proportion of fat mass to total fat mass, DXA has emerged as a potential diagnostic marker, revealing significant differences between patients with lipedema and healthy controls. This differentiation is maintained irrespective of body mass index (BMI) and regional fat distribution.^29^

Finally, a histological clinical examination may be requested, in which tissue affected by lipedema may reveal some distinctive characteristics. These include morphological changes such as increased epidermal thickness, increased adipocyte size indicative of adipocyte hypertrophy, a tendency towards increased fibrosis in adipose tissue, infiltration of immune cells, especially M2 macrophages, and no significant changes in lymphatic vessel coverage, either in terms of number or size, which are important characteristics revealed by histological clinical examination.^39^

The relevance of these additional tools for diagnosing lipedema is crucial in clinical practice, especially when the patient presents concurrent conditions such as lymphedema and/or obesity.

### Types of treatment

Currently, there are no curative treatments for lipedema; approaches aim to improve or alleviate symptoms, prevent disease progression, address complications, and manage patient expectations to provide better physical and psychological quality of life.^20,22-26^

There are two main approaches to treating lipedema: conservative treatment and surgical intervention. Conservative treatments include patient education, weight control, dietary modification, complex decongestive therapy, and electrophysical agents. Surgical interventions primarily consist of liposuction.^20,26^

## CONSERVATIVE TREATMENT

### Lifestyle changes and physical activity

Individuals with lipedema often have a high body mass index and are classified as overweight or obese. Therefore, encouraging weight loss and preventing obesity are crucial steps in treating lipedema and recommendations for physical activity are among the initial conservative measures adopted.^5,6^

The physical activities recommended include swimming, multijoint exercises on vibrating platforms, elliptical machines, stationary bikes, and walking. Additionally, muscle strength exercises, particularly focusing on postural and pelvic floor muscles, are indicated, since individuals with lipedema may have up to 30% less muscle strength than normal individuals.^40,41^

### Nutritional education

Weight gain in lipedema can stem from increased caloric intake and decreased energy expenditure. Considering the importance of physical activity, a healthy dietary approach should be incorporated. Research by Keith et al.^42^ suggested the ketogenic diet as a therapeutic approach for lipedema, involving carbohydrate restriction to induce ketosis, promoting fat utilization for energy and generating ketones. This metabolic strategy positively impacts hormonal processes related to lipid and energy regulation, aiding weight reduction and mitigating lipedema symptoms such as adipose deposition, pain, and inflammation.

The modified Mediterranean diet is another proposed dietary approach characterized by hypocaloric intake and enrichment with antioxidants and anti-inflammatory agents from healthy foods while restricting sugars and processed fats. Studies by Di Renzo et al.^43^ revealed significant weight reduction in the upper and lower limbs of individuals with lipedema following a modified Mediterranean diet compared with controls.

In addition to dietary strategies, supplements can be considered as part of the treatment plan. Various supplements aim to optimize metabolism, promote fat burning, increase lean mass, and facilitate weight loss. However, supplement selection should be personalized, considering individual needs and treatment response, particularly in cases of lipedema where fat may be resistant to dietary therapy and physical activity. Despite advances in dietary methods, specific evidence-based diets for lipedema patients are lacking, because of the limited number of randomized clinical trials with significant samples.^44^

### Physiotherapeutic approaches

Physiotherapeutic intervention plays a crucial role in the conservative treatment of lipedema through either manual therapies or the use of electrophysical agents. The main focus lies in optimizing lymphatic function, improving quality of life, reducing the volume of the affected limb, relieving pain, improving mobility and muscle strength, providing skin care, and preventing complications and disease progression.^45,46^

### Complex decongestive therapy

Complex decongestive therapy (CDT) is currently considered the gold standard for treating lymphedema; however, its benefits can also be extended to individuals with lipedema. CDT is a combination of four therapeutic components: manual lymphatic drainage (MLD), compression therapy through multilayer bandaging or use of compression garments, prescription of myolymphokinetic exercises, and skin care.^47-49^

In a study conducted by Atan and Ozdemir,^48^ the impacts of exercise-based rehabilitation were examined in patients with severe lipedema, both in combination with CDT and with exercise alone. The results revealed improvements in all the measures assessed after the intervention, including limb volume, the 6-minute walk test, the visual analog pain scale, the Fatigue Severity Scale, the Beck Depression Inventory, and the Short Form Health Survey-36 (SF-36). However, when differences between treatment methods were analyzed, it was observed that CDT, when administered in conjunction with exercise, resulted in significant improvements, including reductions in limb volume, pain relief, and improvements in physical function.

Donahue et al.^49^ highlighted the positive effects of CDT in the early stages of lipedema. The therapy showed efficacy for improving touch hypersensitivity. These results were objectively evidenced by the reduction in tissue sodium deposition, which was assessed through magnetic resonance imaging. Sodium, considered a biomarker associated with inflammation and pain in the legs of individuals with lipedema, was present at low levels, a fact that the authors attributed to the physiological action of MLD, a component of CDT.^50-54^

### Compression therapy

Compression therapy plays an essential role in the clinical treatment of lipedema, as signs of lymphatic dysfunction are present even in the earliest stages of lipedema.^24^ Compression therapy increases hydrostatic pressure by exerting pressure on blood and lymphatic vessels, optimizing venous and lymphatic return.^50,55-57^

According to Paling and Macintyre,^56^ compression therapy should be initiated in the early stages of the disease. Simultaneously, compression triggers proprioceptive stimuli by pressing on receptors in the skin, muscles, and joints. These stimuli generate signals that reach the central nervous system, providing support and stability.^56-59^

Compression therapy can be applied using bandages, stockings, or compression garments tailored to the stage of lipedema. This approach supports soft tissue, reducing mechanical impairment of movement caused by friction between skin lobes and improving mobility. Additionally, it contributes to reducing tissue hypoxia.^50,54-57^

The International Lipedema Association (ILA) states that compression therapy plays a central role in treating lipedema. Its purpose is to reduce edema and combat low-grade inflammation, alleviate pain, and mitigate mobility restrictions associated with increased adipose tissue.^55-60^

According to the American guidelines proposed by Herbst et al.,^61^ compressive stockings of 10–20 mmHg should be prescribed for grade 1 lipedema, whereas stockings of 20–40 mmHg may be prescribed for grades 2 and 3. Patients with lipo-lymphedema, grade 4, should be prescribed multilayer compression bandaging.

Despite the benefits associated with use of compression therapy, it is important to note that it may present difficulties in adaptation because of the leg shapes of lipedema patients. Therefore, guidance on proper use and the importance of compression therapy should be carefully explained in detail to the patient.^53-55,57,58^

### Electrophysical agents

Physical therapy offers a variety of electromedical resources, many of which have been proposed for conservative treatment of lipedema. The goal is to promote lipolysis and/or adipose tissue apoptosis, reduce edema, alleviate pain, and decrease low-grade inflammation.^59-61^

Resources such as radiofrequency, therapeutic ultrasound, focused ultrasound, and cryolipolysis, among others, benefit superficial adipose tissue under normal conditions. However, no controlled or randomized clinical studies in patients with lipedema have proven their safety and clinical efficacy. Clinical reports indicate a possible inflammatory effect caused by these technologies, leading to paradoxical adipose tissue hyperplasia in lipedema.^60-62^

### Intermittent pneumatic compression therapy

Szolnoky et al.^50^ compared intermittent pneumatic compression therapy and CDT for treating patients with lipedema. The results indicated that both approaches were equally effective in reducing limb volume and capillary fragility, improving symptoms. In Wright’s study, a group undergoing pressotherapy achieved twice the volume reduction compared with more elastic materials. Further research with broader samples is still needed to validate and strengthen these findings.^51,58,59^

### Low-frequency vibrotherapy

Schneider^60^ analyzed the effects of low-frequency, vertical, and gentle pulse vibratory therapy on an adjustable table in thirty female patients diagnosed with stage II and III lipedema. This therapy involved a combination of manual lymphatic drainage (DLM) and physical vibration, with a low frequency ranging from 15 Hz to 42 Hz. There were substantial reductions in ankle, calf, and thigh circumferences in the combined treatment group compared with those in the group receiving only DLM.

Therefore, vibrotherapy may provide additional benefits due to reduced interstitial pressure and opening of lymphatic capillaries, improving lymphatic flow.^47,60^

### Manual therapy

Presence of fibrosis is notably evident from stage 1 of lipedema and poses significant challenges in the context of treatment. Faced with this complexity, manual therapies have emerged as an intervention aimed at mobilizing and releasing fibrous adhesions.^61,62^

A pilot study conducted by Herbst et al.^61^ demonstrated significant improvements in the structure of subcutaneous adipose tissue, perception of leg function, and volume. Despite the absence of a significant impact on body shape, DXA scans indicated a trend towards reduced limb and trunk fat.

Another study conducted by Ibarra et al.^62^ examined manual therapy with subcutaneous adipose tissue in women with lipedema and Dercum’s disease. After twelve sessions over four weeks, ultrasound revealed improvements in fat structure and fascia.

These findings point to the potential efficacy of manual therapy as an adjunct in treating lipedema.

### Shock waves

Extracorporeal shock wave therapy (ESWT) has emerged as a prominent modality in physiotherapy practice, especially in the treatment of lymphatic and aesthetic dysfunctions. In adipose tissue, ESWT appears to modulate the metabolism of adipocytes, promoting autophagic lipolysis through the apoptotic pathway. Given these findings, its application for treating lipedema has been proposed.^63-65^

Siems et al.^66^ evaluated patients with lipedema and cellulite who were receiving complex physical decongestive therapy (CPDT) daily in conjunction with ESWT twice a week. The results indicated that this combination of therapies contributed to a reduction in oxidative stress and a consequent reduction in the levels of fibrogenic cytokines, with improvements in skin biomechanical properties.

Michelini et al.^67^ investigated the effects of a treatment involving unfocused and radial ESWT, mesotherapy, and application of Kinesio tape in patients with stage II lipedema. The results revealed reductions in pain and circumference measurements with a decrease in thickness, improvement in echographic pattern, and increased elasticity of subcutaneous adipose tissue, resulting in a positive impact on quality of life.

### Photobiomodulation

Studies by Baxter et al.,^68^ Chen et al.,^69^ and Chiu et al.^70^ demonstrated that combining photobiomodulation with compressive physical therapy to treat lymphedema significantly enhanced patient outcomes. With proven benefits in lymphedema, there is biological plausibility regarding the effects of photobiomodulation on lipedema.

Recent research has improved understanding of photobiomodulation in the mitochondrial pathway. Evidence suggests that photobiomodulation, with red and infrared wavelengths, stimulates a cascade of cellular events. This physiological effect can be extremely beneficial in the conservative treatment of lipedema when combined with pain relief and fibrosis modulation. However, further research is needed to assess the extent of this therapy and its effectiveness in the specific treatment of lipedema.^71-73^

### Drug treatment and supplements

Medication in lipedema management is generally aimed at relieving associated symptoms such as pain and inflammation. Although there are no medications specifically approved for lipedema treatment, nonsteroidal anti-inflammatory drugs (NSAIDs) can be used to control acute pain for short periods and curcumin can be used for longer periods. Additionally, agents that improve microcirculation and lymphatic drainage, such as diosmin and hesperidin (flavonoids), may be prescribed to improve symptoms of discomfort and swelling. Metformin appears to have an antifibrotic effect and is recommended in the latest American guidelines.^9,13,26,43^

Herbal medicines and natural supplements have also been explored for lipedema treatment, aiming to reduce swelling and improve blood and lymphatic circulation. Substances such as Centella asiatica are recognized for their venotonic properties and improved skin elasticity, which may help reduce the sensation of leg heaviness and fatigue.^9,13,26,43^

## SURGICAL TREATMENT

### Liposuction

Liposuction is the only therapeutic approach capable of removing abnormal adipose tissue. It can be indicated for cosmetic or functional purposes. When combined with conservative treatments, this approach can achieve patient satisfaction with the appearance of their limbs and quality of life.^27,29,74,75^

Typically, liposuction is performed using the tumescent technique (TA), in which a saline solution with adrenaline and local anesthetics is infused into the area to be treated. Using thin cannulas connected to a suction device, fat can be removed circumferentially from the affected limb through multiple small incisions. At the end of the procedure, these incisions may be left open for healing by secondary intention, aiming to reduce seroma formation.^74,75^

Patients at advanced stages may have large amounts of deposited adipose tissue, and it may be necessary to remove large volumes to achieve the desired results. Federal Medical Council Resolution 1711/03 establishes a safety parameter of removal of a maximum of 7% of body weight in aspirated volume. Thus, depending on the degree and distribution of fat, more than one procedure may be necessary for complete treatment. Planning and the number of surgical steps should ideally be discussed before the first procedure.^74-78^

Many patients may experience increased skin laxity in the areas treated to remove excess fatty tissue. Thus, combining technologies such as lasers and radiofrequency may contribute to greater skin retraction. However, no studies have reported their efficacy or potential adverse effects in this population.^74,75^

The postoperative period requires attention from a specialized multidisciplinary team. Compression therapy should be initiated as early as possible. Significant drainage of serous fluid from the incisions is expected and requires frequent dressing changes and bandaging. These are procedures with the potential to limit patient ambulation, and use of chemoprophylaxis for venous thromboembolism should be considered.

Although liposuction has been more recently incorporated into the therapeutic arsenal for lipedema, there is evidence regarding its use as a treatment modality in this population. Peprah and MacDougall^74^ conducted a systematic review of the efficacy of liposuction for treating this disease. The main results revealed a significant reduction in thigh circumference, ranging from 6 to 8 cm; an average decrease of 6.9% in leg volume; notable improvements in mobility capacity; a reduction in spontaneous pain and sensitivity to pressure; edema reduction; bruising reduction; decreased sensation of tension; and significant improvement in quality-of-life impairment scores.

A cross-sectional study^75^ investigated the efficacy of liposuction for treating lipedema, focusing on the treatment’s impact on pain reduction. The study concluded that liposuction demonstrated efficacy for reducing pain associated with lipedema (from 6.99 to 2.24), regardless of the stage of the disease, suggesting that this intervention could be part of a multimodal treatment approach to improve patients’ quality of life.

Despite liposuction being considered traumatic surgery, studies indicate low complication rates, comparable to rates reported in larger cohorts of patients without lipedema (1% bleeding, 4% erysipelas, and 4.5% wound infection). However, the importance of post-liposuction physiotherapeutic monitoring has been highlighted, focusing on rehabilitation and preventing complications.^74,75^

Studies have shown that liposuction can be considered a promising therapeutic approach for treating lipedema. However, current evidence is still limited to establish liposuction as the gold standard for treatment. Most available studies are observational, with small sample sizes and a lack of standardization of surgical protocols. There is therefore a clear need for more robust future studies, such as randomized controlled trials, to assess the long-term efficacy and safety of liposuction more accurately. Additionally, it is essential to investigate potential complications and adverse effects associated with this procedure to ensure patient safety. Thus, liposuction should be considered part of a multimodal treatment approach, complementing nonsurgical interventions.

### Surgical treatment of varicose veins associated with lipedema

Treating varicose veins before lipedema surgery is crucial to minimize the risk of postoperative complications, such as bleeding, hematoma, and phlebitis. Moreover, varicose veins may indicate a predisposition to poor blood circulation, increasing the risk of deep vein thrombosis (DVT), especially in leg surgeries, according to the Caprini score. Therefore, proper evaluation and treatment of varicose veins are essential before surgery for lipedema.^23,26^

Treatment strategies for varicose veins may include noninvasive methods such as wearing compression stockings, specific exercises, lifestyle changes, minimally invasive procedures (sclerotherapy, radiofrequency, or laser ablation), and phlebectomy.^41,48^

These interventions aim to improve blood flow and reduce venous pressure. Improved venous circulation may also facilitate postoperative recovery. Therefore, an integrated approach that includes prior varicose vein treatment not only enhances the safety of lipedema surgery but also reinforces the importance of thorough vascular assessment as part of surgical planning, ensuring safer and more effective intervention.^23^

## PATIENT EDUCATION

Evidence from studies reveals that individuals with lipedema experience challenges in psychological adjustment, amplified deficits in physical and social skills, and increased anxiety and depression. Prevalent symptoms such as pain and sensitivity indicate potential negative impacts on health-related quality of life.^79,80^

Therefore, in conjunction with conservative and surgical approaches supported by a multidisciplinary team, treating patients with lipedema should incorporate guidance and education about the chronic and progressive nature of the condition, which has no definitive cure.^79,80^

Despite the advances made in understanding lipedema, it is crucial to highlight the ongoing need for further research.

This article outlines the evolution of understanding of lipedema from its initial description to contemporary therapeutic strategies. Given the absence of a definitive cure, conservative approaches play a crucial role, with surgical treatment being the last valid option when these measures prove insufficient to provide significant improvements in symptoms and quality of life.

Despite the advances achieved in understanding lipedema, challenges persist, especially in refining physiological and diagnostic issues. The complexity of the disease, which is often underdiagnosed, underscores the urgency of further research to deepen understanding of this unique condition, aiming to develop more promising treatment strategies.

## CONCLUSION

Lipedema is a chronic condition that affects adipose tissue. Treatment is focused on conservative strategies and, in some cases, surgical interventions, aiming to alleviate symptoms and improve function, thus yielding better quality of life. Despite advances in understanding and treatment, lipedema presents persistent challenges. Pathophysiology and diagnosis require ongoing attention and further research is essential for significant advances that aim to achieve more effective management of this complex condition.
